# Retraction

**DOI:** 10.1111/jcmm.17756

**Published:** 2023-05-07

## Abstract

Ma, H., Li, S.‐Y., Xu, P., Babcock, S.A., Dolence, E.K., Brownlee, M., Li, J. and Ren, J. (2009), Advanced glycation endproduct (AGE) accumulation and AGE receptor (RAGE) up‐regulation contribute to the onset of diabetic cardiomyopathy. Journal of Cellular and Molecular Medicine, 13: 1751–1764. https://doi.org/10.1111/j.1582‐4934.2008.00547.x.

The above article, published online on 13 October 2008 in Wiley Online Library (wileyonlinelibrary.com), has been retracted by agreement between, the journal Editor in Chief, Stefan Constantinescu, The Foundation for Cellular and Molecular Medicine, and John Wiley and Sons Ltd. The retraction has been agreed as requested by The University of Wyoming. Following a review, a university investigation committee found evidence of data irregularities and image reuse in Figure 5 that significantly affect the results and conclusions reported in the manuscript. As a result, the article's conclusions can no longer be considered valid.

Ma, H., Li, S.‐Y., Xu, P., Babcock, S.A., Dolence, E.K., Brownlee, M., Li, J. and Ren, J. (2009), Advanced glycation endproduct (AGE) accumulation and AGE receptor (RAGE) up‐regulation contribute to the onset of diabetic cardiomyopathy. Journal of Cellular and Molecular Medicine, 13: 1751–1764. https://doi.org/10.1111/j.1582‐4934.2008.00547.x.

The above article, published online on 13 October 2008 in Wiley Online Library (wileyonlinelibrary.com), has been retracted by agreement between, the journal Editor in Chief, Stefan Constantinescu, The Foundation for Cellular and Molecular Medicine, and John Wiley and Sons Ltd. The retraction has been agreed as requested by The University of Wyoming. Following a review, a university investigation committee found evidence of data irregularities and image reuse in Figure 5 that significantly affect the results and conclusions reported in the manuscript. As a result, the article's conclusions can no longer be considered valid.

